# Comparison of a dorsolateral approach and a dorsomedial approach to access the medial malleolus of the distal tibia in horses

**DOI:** 10.1111/vsu.14241

**Published:** 2025-03-10

**Authors:** Margherita Guerra, Lauren V. Schnabel, Carrie C. Jacobs

**Affiliations:** ^1^ Department of Clinical Sciences, College of Veterinary Medicine North Carolina State University Raleigh North Carolina USA

## Abstract

**Objective:**

To determine the utility of a dorsolateral arthroscopic approach to the tarsocrural joint (TCJ) to examine and surgically access the medial malleolus (MM) and compare this to the standard dorsomedial approach to the MM.

**Study design:**

Experimental cadaver study.

**Animals:**

Six pelvic limbs from four adult horses.

**Methods:**

Arthroscopic examination of the dorsal aspect of the TCJ was performed followed by the dorsomedial and dorsolateral surgical approaches to the MM, in randomized order on cadaver limbs (*n* = 6). The dorsomedial approach involved placing the arthroscope and instrument in the dorsomedial pouch. The dorsolateral approach involved placing the arthroscope dorsolaterally and the instrument dorsomedially. Identification and surgical access grades for the MM were assessed and recorded.

**Results:**

Using the dorsomedial approach, identification grades were excellent for the MM and surgical access to the MM was excellent or good in all limbs. Using the dorsolateral approach, identification and surgical access grades for the MM were excellent in all limbs. Interference between the arthroscope and instrument only occurred during the dorsomedial approach. The view of the axial aspect of the MM was improved with the dorsolateral approach.

**Conclusion:**

The dorsolateral approach allowed identification and surgical access to the MM and provided an improved view of the axial aspect of the MM. No interference between the instrument and arthroscope was encountered.

**Clinical significance:**

The dorsolateral arthroscopic approach to the TCJ can be used for debridement of MM OCD lesions.

## 1 INTRODUCTION

Osteochondrosis dissecans (OCD) is a common developmental orthopedic disease affecting horses and is found with the highest frequency in the tarsocrural, metacarpo−/metatarsophalangeal, and femoropatellar joints.^1^ OCD of the tarsocrural joint (TCJ) can occur in one or multiple predilection sites within the same joint.^2^, ^3^, ^4^ The most common predilection sites within the TCJ are the distal intermediate ridge of the tibia (DIRT), lateral trochlear ridge (LTR), and medial malleolus (MM).^5^ Arthroscopy is the gold standard for treatment of OCD and is also used diagnostically in the evaluation of cases with idiopathic joint effusion where OCD is suspected but difficult to diagnose radiographically.^6^, ^7^, ^8^

Arthroscopic exploration of the dorsal aspect of the TCJ involves creating a dorsomedial portal for placement of the arthroscope with a dorsolateral instrument portal to access lesions of the LTR and DIRT.^6^ Lesions of the MM are often located on the axial aspect of the malleolus with the recommended surgical approach placing both the arthroscope and instrument portals in the dorsomedial aspect of the TCJ.^6^ The curvature of the malleolus, the decreased ability for triangulation due to the proximity of the instrument and arthroscope, and interference of the instrument and the arthroscope can make surgical access of MM lesions challenging. A surgical approach using a dorsolateral arthroscope portal and a dorsomedial instrument portal has been previously mentioned for accessing lesions of the MM, but is not the recommended or commonly reported surgical approach for these lesions.^5^, ^6^ Additionally, the technique, the view of the MM, and any potential advantages of this approach are not well described. Therefore, our objective was to determine the utility of a dorsolateral arthroscopic approach to the tarsocrural joint (TCJ) to examine and surgically access the MM and compare this to the standard dorsomedial approach to the MM. We hypothesized that a dorsolateral approach would allow surgical access to the MM and that this approach would alleviate instrument interference and improve the view of the axial aspect of the MM compared to the dorsomedial approach.

## 2 MATERIALS AND METHODS

Six pelvic limbs from four adult horses euthanized for reasons unrelated to the study were transected at the mid‐tibia and used for this study. The limbs were frozen at −20°C and thawed for 24 h at room temperature prior to use. The limbs were positioned similarly to how they would be positioned in dorsal recumbency. To achieve this, the transected tibia was grasped and secured to a surgical table with a bench clamp. A hobble was placed around the pastern and secured to a hoist to allow flexion and extension of the limb by the surgeon. All arthroscopies were performed by a surgical resident (MG) under the supervision of a diplomate of the American College of Veterinary Surgeons (CCJ).

Routine distension of the TCJ was performed with 60 mL of saline. For all limbs, the arthroscope was first placed in the standard dorsomedial portal.^6^ With the limb in extension, a 5‐mm skin incision was made using a No. 15 blade just medial to the extensor tendons and distal to the MM. A No. 11 blade was used to extend the portal through the joint capsule. The arthroscopic sleeve and conical obturator were inserted, the obturator was replaced with a 3‐mm diameter, 30° forward arthroscope (Arthrex, Naples, Florida) available for research use and entry into the joint was confirmed by identification of cartilage. Following routine exploration of the joint, both the dorsomedial approach to the MM and dorsolateral approach were performed, as described below, with the approach performed first randomized for each limb. For each approach, the identification and surgical accessibility of the MM was graded as described below.

### 2.1 Dorsomedial approach to the MM

For creation of the dorsomedial instrument portal to approach the MM, the limb was placed in extension. Due to the axial location of the arthroscope, an instrument portal was made 1 cm abaxial and 1 cm distal to the arthroscopic portal. An 18‐gauge needle was used at the proposed portal site to confirm that the MM could be accessed prior to making the incision. Identification and surgical access grades for the MM were performed with the limb maintained in extension as this is the position used for the described dorsomedial approach to the MM.^6^

### 2.2 Dorsolateral approach to the MM

For the dorsolateral approach, the portal into the dorsolateral pouch of the TCJ was created with the limb maintained in flexion. The location of the portal was identified using an 18‐gauge needle passed horizontally over the LTR in the direction of the DIRT. A No. 11 blade was used to make an incision through the skin and joint capsule at the location of the needle, and a cannula and conical obturator were introduced into the dorsolateral joint pouch. The obturator was exchanged with the arthroscope and the dorsomedial portal was used as the instrument portal. A switching stick was available for use, but not needed. The joint was explored and MM identification and surgical accessibility graded with the instrument and arthroscope in this position.

### 2.3 Grading

The ability to identify the MM (yes/no) and surgically access it with an arthroscopic probe (yes/no) was determined for each approach. Both the identification and surgical access of the MM was then graded as poor, good, or excellent. If the MM could not be seen and/or the arthroscopic instrument could not touch any part of the structure, no grade was given. Poor indicated that the MM was visible but the entire MM could not be seen or that the MM could be touched with the probe but position of the instrument prevented surgical access or movement of the instrument to a theoretical OCD lesion of the distal MM. This theoretical OCD lesion was considered to extend from the distal MM up to the midpoint of the axial aspect of the MM. Good indicated that the entire MM could be evaluated and this view allowed visualization of the instrument to a theoretical OCD lesion of the distal MM, but the view was not ideal. Regarding surgical accessibility, if the instrument could access a theoretical OCD lesion of the distal MM but the surgeon could not manipulate the instrument to all parts of the distal MM, this was graded as good. Excellent indicated that the entire MM was visible and the surgeon had the ideal view for surgical manipulation of a theoretical OCD lesion of the distal MM. Regarding surgical accessibility, if the instrument could access a theoretical OCD lesion of the distal MM and surgeon could move the instrument to all parts of the distal MM, this was graded as excellent. Challenges such as instrument or soft tissue interference, difficulty entering the joint, or any iatrogenic damage to the joint were recorded.

## 3 RESULTS

### 3.1 Dorsomedial approach to the MM

**FIGURE 1:**
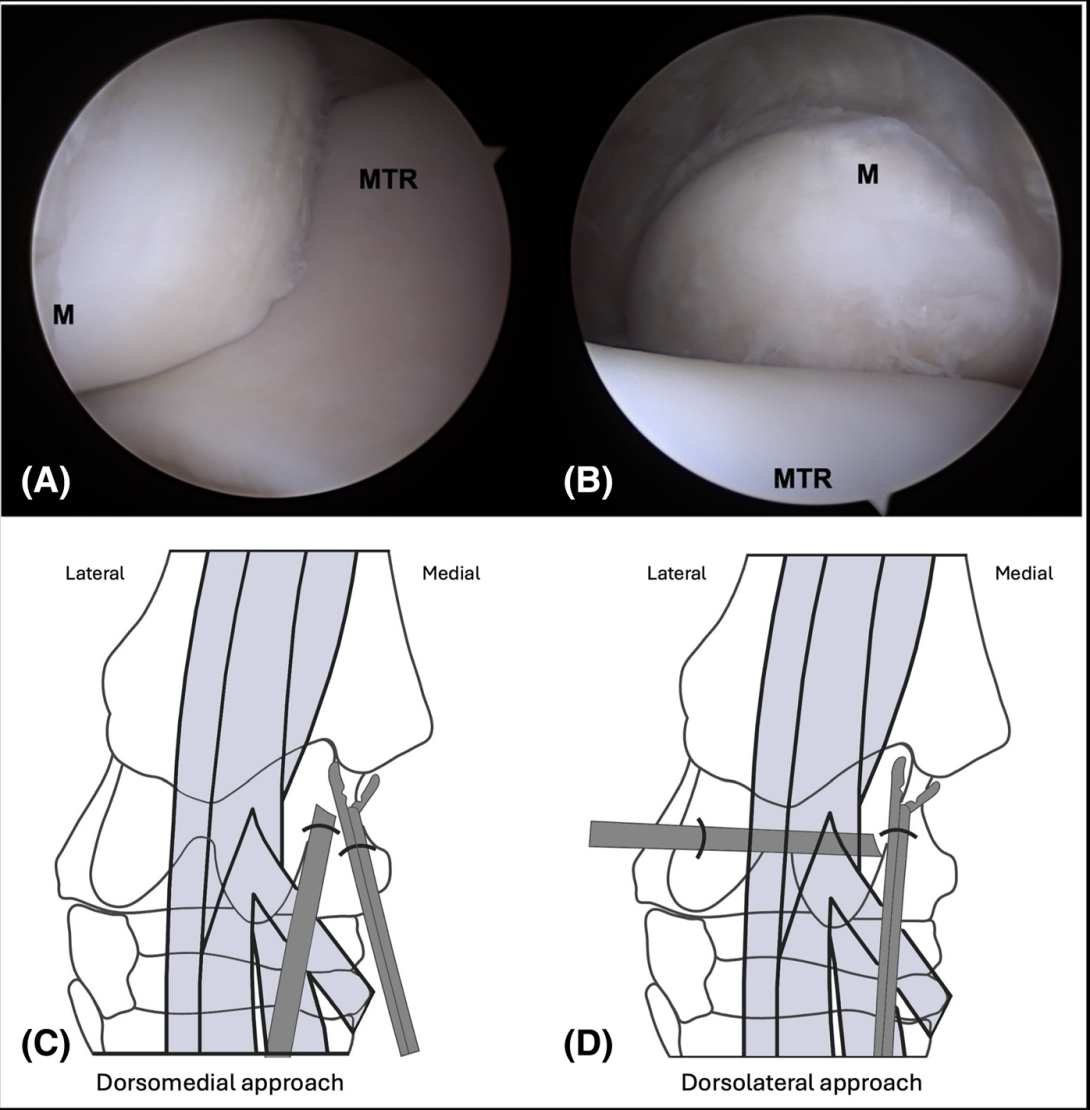
The medial malleolus of the same limb viewed from (A) a dorsomedial approach and from (B) the dorsolateral approach. Diagram of the positions of the arthroscope and instrument for (C) the dorsomedial and (D) dorsolateral approach are shown below the corresponding arthroscopic image. M, medial malleolus; MTR, medial trochlear ridge. Figure created with BioRender.com.

**FIGURE 2:**
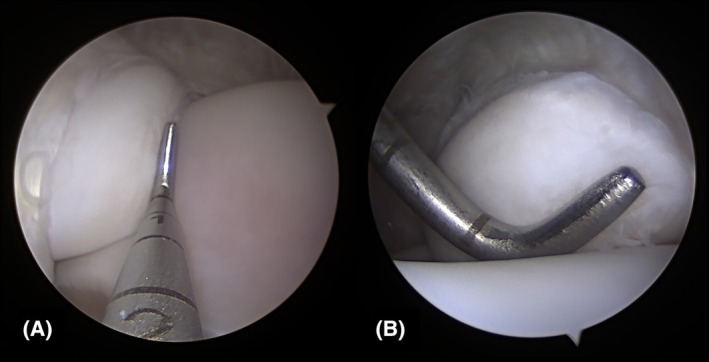
The medial malleolus viewed from (A) a dorsomedial approach and from (B) the dorsolateral approach with a probe.

With the arthroscope placed in the dorsomedial pouch of the TCJ, identification grades for the MM were excellent in all limbs (Table 1). Using this approach, the abaxial and distal portion of the MM were highlighted (Figure 1A). When the instrument was placed in the dorsomedial pouch, surgical access to the MM was graded as excellent or good in all limbs (Table 1). Interference between the arthroscope and instrument was recorded in 3/6 limbs using this approach. The close working distance between the instrument and arthroscope with the dorsomedial approach is apparent in Figure 2.

**TABLE 1:** Identification and surgical accessibility grades for the dorsomedial and dorsolateral approaches for the medial malleolus.

	Dorsomedial approach				Dorsolateral approach			
	Accessible	Excellent	Good	Poor	Accessible	Excellent	Good	Poor
Identification	6/6	6	‐	‐	6/6	6	‐	‐
Surgical access	6/6	5	1	‐	6/6	6	‐	‐

### 3.2 Dorsolateral approach to the MM

With the arthroscope placed in the dorsolateral pouch of the TCJ, identification grades for the MM were excellent in all limbs (Table 1). Using this approach, the proximal and axial aspects of the MM were highlighted (Figure 1B). No interference was noted between the arthroscope and instrument in any limb. The improved working distance between the instrument and arthroscope in the dorsolateral approach is seen in Figure 2. With the dorsolateral approach, surgical access grades for the MM were excellent in all limbs (Table 1).

## 4 DISCUSSION

Based on our results, both identification and surgical access to the MM were achieved using a dorsolateral approach to the TCJ. Interference between the instrument and arthroscope was not experienced using a dorsolateral approach and the view of the axial aspect of the MM was improved using a dorsolateral approach compared to a dorsomedial approach.^6^ Therefore, this information supports our hypothesis.

Within the TCJ, OCD lesions most commonly occur at one predilection site but have been reported at multiple predilection sites within the same joint.^2^, ^3^, ^4^ In a study of 69 joints with lesions of the MM, 28 (40.5%) joints had additional OCD lesions, most located at the DIRT (26/28, 93%).^2^ Using the conventional approach to address DIRT, LTR, and MM OCD, a total of three portals are usually required to achieve surgical access to all TCJ OCD predilection sites. While switching arthroscope and instrument portals is commonly performed in arthroscopy, doing so for debridement of MM lesions is not well described. By switching the dorsomedial arthroscope and dorsolateral instrument portals typically used for the approach for OCD of the DIRT/LTR, the dorsolateral arthroscopic approach provided appropriate identification of and surgical access to the MM. By using this approach, all three TCJ OCD predilection sites should be surgically accessible using only two portals. Ideally, arthroscopy is performed using as few portals as possible as creation of additional portals can cause loss of joint distension, decreased visibility, and potentially impaired surgical accessibility. Additionally, an increased number of surgical incisions would presumably increase the risk for infection and potentially septic arthritis, particularly in the TCJ where a higher likelihood of postoperative infection has been reported.^9^ Therefore, the use of an arthroscopic approach that allows complete exploration of the dorsal joint pouch and optimizes surgical access to TCJ OCD predilection sites while limiting portal number is potentially advantageous.

Additionally, accessing the MM with a dorsolateral approach improved the view of the axial aspect of the MM. Lesions of the MM are often located axially and may extend along the plantar and axial border of the MM.^6^ Therefore, using the dorsolateral approach may provide improved identification and surgical accessibility of axially located OCD lesions of MM. In addition to allowing surgical access to MM OCD lesions, switching arthroscope and instrument portals improves the overall exploration of the joint. While this was not evaluated in this study, the use of a dorsolateral arthroscopic portal has been described to evaluate the lateral collateral ligaments of the tarsocrural joint.^10^

While both the dorsolateral approach and the dorsomedial approach had similar grades for surgical access to the MM, it is important to note that interference between the arthroscope and the instrument only occurred with the dorsomedial approach. Interference is due to the proximity of the arthroscope and instrument portals and limited triangulation. This is avoided with the use of a dorsolateral arthroscopic portal and a dorsomedial instrument portal. This places the arthroscope and instrument portals at a greater distance from each other compared to their position using the dorsomedial approach in which the two are commonly placed 1–2 cm apart (Figure 2).

**FIGURE 3:**
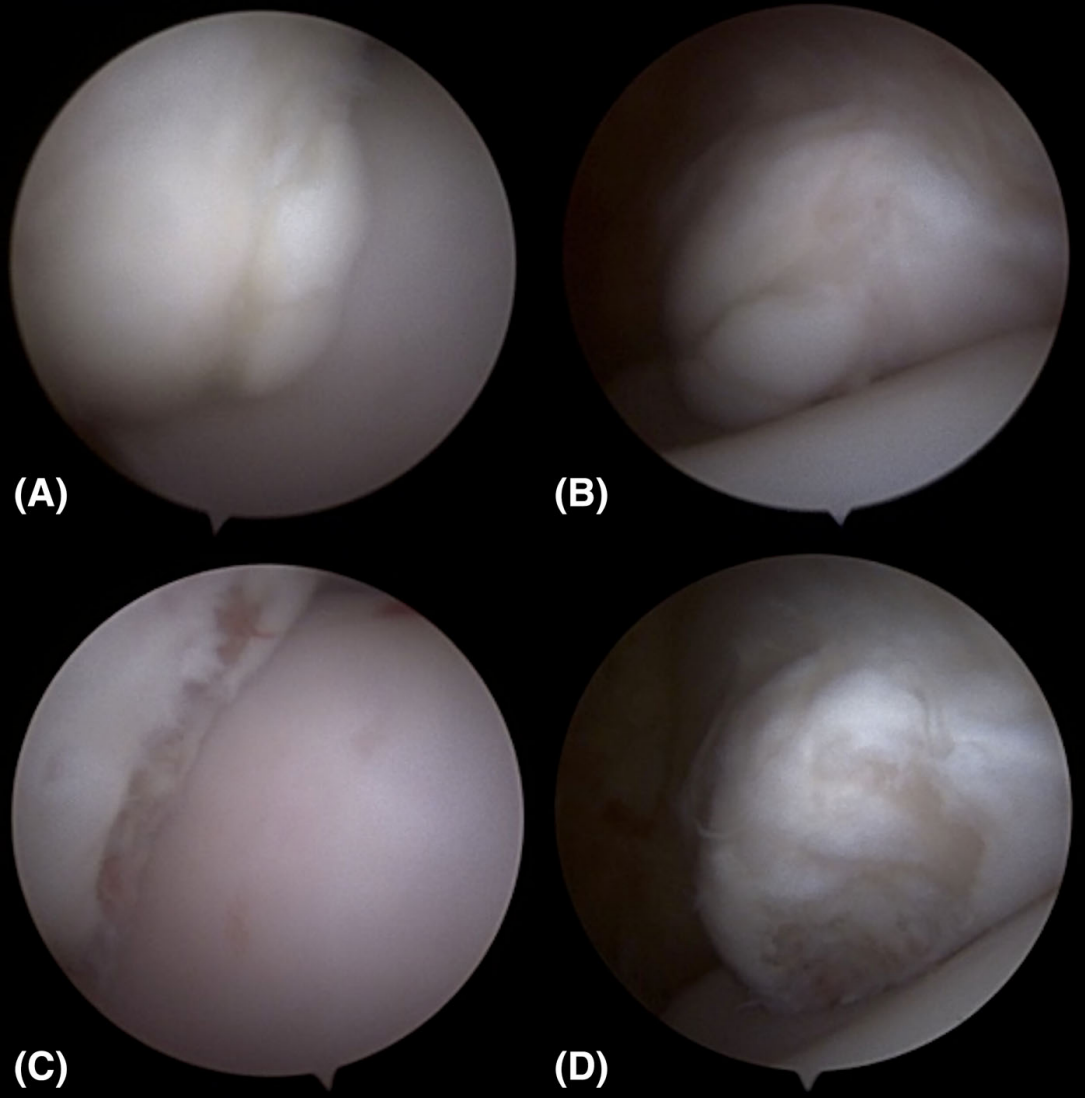
Appearance of a medial malleolus osteochondrosis dissecans lesion from (A) the dorsomedial approach and (B) the dorsolateral approach and following its removal as viewed from (C) the dorsomedial approach and (D) the dorsolateral approach.

When multiple OCD lesions are identified either on radiographic evaluation or upon arthroscopic exploration, the authors prefer to start with the arthroscope placed in a dorsomedial portal and an instrument placed in a dorsolateral portal. After lesions of the DIRT or LTR have been addressed, the arthroscope is placed in the dorsolateral portal and the instrument placed through the dorsomedial portal to address lesions of the MM. Figure 3 shows the views of the MM from each approach with a clinical OCD lesion. Using the dorsomedial arthroscopic approach in addition to switching the arthroscope and instrument portals, multiple OCD lesions can be addressed using only two portals.

While the dorsolateral approach uses the same portals as described for accessing the DIRT and LTR, planning of these portals for successful use for MM debridement is crucial. When initially making the dorsolateral portal, it is important to ensure the portal is placed dorsally such that when the arthroscope is switched it can pass easily over the LTR. This is crucial to allow identification of the MM without inflicting iatrogenic cartilage damage. It is also recommended to position the initial dorsomedial arthroscope portal as axial as possible (axial to the saphenous vein and abaxial to the extensor tendons) to allow more direct access to the axial aspect of the MM with the instrument once the portals are switched.

While this was not encountered in this study, difficulty entering the joint and unwanted extravasation of fluid can occur when switching the arthroscope and instrument portals, such as what is required for the dorsolateral approach. Switching of the arthroscope in this study was performed with a conical obturator and cannula. A switching stick could have been used for this step to ensure ease of entrance of the arthroscope in the dorsolateral portal. The initial dorsomedial portal is created with the limb in extension. When the limb is flexed to explore the joint, the skin incision moves in relation to the incision into the joint capsule. When this arthroscopic portal becomes an instrument portal, this incongruence may make introduction of an instrument into this portal initially challenging and can cause increased extravasation of fluid. It may be necessary to enlarge the dorsomedial portal to improve the ease of instruments passing into the joint and to prevent unwanted extravasation. In the cadaver study, the only instrument passed through the dorsomedial portal was a probe, which is of similar diameter to the arthroscope, so enlargement of the portal was not needed. If difficulty is encountered with the dorsolateral approach, the dorsomedial approach to the MM can still be performed.

There were several limitations with this study, the first being the use of cadaver limbs without OCD lesions. Surgical access was inferred based on the ability to reach the distal aspect of the MM with an arthroscopic probe followed by manipulation of an arthroscopic probe in this area. The simulated surgical access was not able to account for the positioning of fragments in relation to the working components of instruments (jaws of rongeurs, angle of curettes), and may have overrepresented the ability for surgical manipulation. Scoring of the cadaver limbs was not blinded and this innately introduces bias to the scoring. Blinding was not possible as even when assessing images or videos, the angle and view of the anatomy indicates the approach used. While the dorsolateral approach is used by the authors in clinical cases of MM OCD, further study on the use of this approach in clinical cases is needed.

In conclusion, the dorsolateral approach provided for good identification and surgical access to the MM. Based on these findings, the dorsolateral approach can be considered as an alternative approach to debride OCD of the MM, particularly in cases with OCD lesions in multiple locations. Further investigation for the utility of this approach in clinical cases is warranted.

## AUTHOR CONTRIBUTIONS

Guerra M, DVM: Contributed to study design, manuscript preparation, editing, submission, and performed cadaver surgeries. Schnabel LV, DVM, PhD, DACVS (Large Animal), DACVSMR: Contributed to study design, manuscript preparation, editing, and submission. Jacobs CC, DVM, DACVS (Large Animal): Contributed to study design, manuscript preparation, editing, submission, and performed cadaver surgeries.

## CONFLICT OF INTEREST STATEMENT

Research reported in this manuscript was supported by the Veterinary Practice Plan account of Carrie C. Jacobs. The authors declare no financial or other conflicts of interest related to this project. This research was presented as a poster at the 2024 American College of Veterinary Surgeons Annual Meeting in Phoenix, Arizona.

## References

[1] van Weeren PR . Osteochondritis dissecans. In: Auer JA , Stick JA , Kummerle JM , Prange T , eds. Equine Surgery. 5th ed. Saunders Elsevier; 2019:1509‐1524.

[2] Bertuglia A , Pallante M , Pagliara E , et al. Determinants of joint effusion in tarsocrural osteochondrosis of yearling standardbred horses. Front Vet Sci. 2024;11. doi:10.3389/fvets.2024.1389798 PMC11303144 39113724

[3] McCoy AM , Ralston SL , McCue ME . Short‐ and long‐term racing performance of standardbred pacers and trotters after early surgical intervention for tarsal osteochondrosis. Equine Vet J. 2015;47(4):438‐444. doi:10.1111/evj.12297 24819047 PMC4229490

[4] Beard WL , Bramlage LR , Schneider RK , Embertson RM . Postoperative racing performance in Standardbreds and Thoroughbreds with osteochondrosis of the tarsocrural joint: 109 cases (1984–1990). J Am Vet Med Assoc. 1994;204(10):1655‐1659. 8050949

[5] McIlwraith CW , Foerner JJ , Davis DM . Osteochondritis dissecans of the tarsocrural joint: results of treatment and arthroscopic surgery. Equine Vet J. 1991;23(3):155‐162. 1884694 10.1111/j.2042-3306.1991.tb02746.x

[6] McIlwraith CW , Nixon AJ , Wright IM . Diagnostic and surgical arthroscopy of the tarsocrural (tibiotarsal) joint. In: McIlwraith CW , Nixon AJ , Wright IM , eds. Diagnostic and Surgical Arthroscopy in the Horse. 4th ed. Mosby Elsevier; 2015:243‐272.

[7] Kadic LIM , Rodgerson DH , Newsom LE , Spirito MA . Description of a rare osteochondrosis lesion of the medial aspect of the distal intermediate ridge of the tibia in seven Thoroughbred horses (2008–2018). Vet Radiol Ultrasound. 2020;61(3):285‐290. doi:10.1111/vru.12843 32020748

[8] Torre T , Toniato M . Osteochondral fragments from the medial malleolus in horses: a comparison between radiographic and arthroscopic findings. Proc Annu Conv AAEP. 1999;45:167‐171.

[9] Olds AM , Stewart AA , Freeman DE , Schaeffer DJ . Evaluation of the rate of development of septic arthritis after elective arthroscopy in horses: 7 cases (1994–2003). J Am Vet Med Assoc. 2006;229(12):1949‐1954. 17173536 10.2460/javma.229.12.1949

[10] Kümmerle JM , Kummer MR . Arthroscopically accessible anatomy of the tarsal collateral ligaments in the horse. Vet Surg. 2013;42(3):267‐274. 23373856 10.1111/j.1532-950X.2013.01100.x

